# Defence priming in Arabidopsis – a Meta-Analysis

**DOI:** 10.1038/s41598-019-49811-9

**Published:** 2019-09-16

**Authors:** Sara M. Westman, Karen J. Kloth, Johannes Hanson, Anna B. Ohlsson, Benedicte R. Albrectsen

**Affiliations:** 1 Umeå Plant Science Centre, Department of Plant Physiology, Umeå University, Umeå Plant Science Centre, Umeå, Sweden; 2 Laboratory of Entomology, Wageningen University, P.O. Box 16, 6700 AA Wageningen, The Netherlands; 3 Department of Industrial Biotechnology, School of Engineering Sciences in Chemistry, Biotechnology and Health (CBH), KTH Royal Institute of Technology, Stockholm, Sweden

**Keywords:** Environmental biotechnology, DNA methylation, Biotic, Environmental impact

## Abstract

Defence priming by organismal and non-organismal stimulants can reduce effects of biotic stress in plants. Thus, it could help efforts to enhance the sustainability of agricultural production by reducing use of agrochemicals in protection of crops from pests and diseases. We have explored effects of applying this approach to both Arabidopsis plants and seeds of various crops in meta-analyses. The results show that its effects on Arabidopsis plants depend on both the priming agent and antagonist. Fungi and vitamins can have strong priming effects, and priming is usually more effective against bacterial pathogens than against herbivores. Moreover, application of bio-stimulants (particularly vitamins and plant defence elicitors) to seeds can have promising defence priming effects. However, the published evidence is scattered, does not include Arabidopsis, and additional studies are required before we can draw general conclusions and understand the molecular mechanisms involved in priming of seeds’ defences. In conclusion, defence priming of plants has clear potential and application of bio-stimulants to seeds may protect plants from an early age, promises to be both labour- and resource-efficient, poses very little environmental risk, and is thus both economically and ecologically promising.

## Introduction

Pesticides are used by farmers globally to protect crops from pests and diseases^1^, and they played important roles in “the green revolution” that brought huge benefits for agriculture and mankind^2^. However, conventional uses of pesticides also have serious drawbacks as they contaminate the environment^3^, cause fatalities^1^, and may foster a false sense of security regarding risks of pest outbreaks^4^. Hence, both national and international authorities, e.g., FAO^5^ and EU^6^ advocate development of alternative strategies.

Current approaches include biotechnological enhancement of plant resistance^7^ and optimization of integrated management efforts^8^. In addition, organismal interactions can be used to promote natural plant resistance interactions and mechanisms^9–13^. There is increasing evidence^14,15^ that plant-associated microorganisms can bolster plants’ resistance to biotic stresses. *Inter alia*, authors including^16,17^ have shown that manipulations of single microorganisms or entire microbiomes can enhance plant health. Plant resistance to biotic stressors can also be strengthened by activating plant defences through innovative uses of non-toxic internal or environmental bio-stimulants^18–20^.

Such stimulants can “prime” plants’ defences, i.e. enhance their responsiveness to biotic stressors^21–23^, thereby potentially offering a sustainable alternative to use of conventional plant protection chemicals^24–27^. Priming initially triggers a minor part of a defence response that increases the plant’s ability to defend itself against future antagonists (for example herbivores or pathogens). Once a plant has been primed, it will defend itself more rapidly, strongly and/or enduringly against subsequent threats. Priming agents may be live organisms (e.g. microorganisms or arthropods), chemicals (e.g. vitamins or plant hormones) or components thereof, and priming can be applied to various tissues and at diverse developmental stages (for example to foliage or roots of mature plants, or to seeds).

Molecular mechanisms underlying priming are far from completely understood, but there is a consensus that primed plants conserve a memory. Two potential mechanisms have been suggested. One involves accumulation of mitogen-activated protein kinases (MPKs). For example, Beckers *et al*.^28^ found that two MPKs (MPK3 and MPK6) accumulate during induction of systemic acquired resistance (SAR) by benzothiadiazole (BTH). Gene expression studies of knockout mutants showed that the kinases remain dormant until the plant is under attack, which initiates accumulation of defence enzymes (Ibid.). The other suggested mechanism is that epigenetic changes in DNA methylation and histone modifications may be carriers of stress memories and triggers of immune responses^29–31^. Reversible histone acetylation may be involved, at least in some cases, according to suggestions by Chen and Tian^32^ and observations of Arabidopsis plants’ responses to BTH^31^. Similarly, Lopez Sanchez, *et al*.^33^ found that hypomethylated Arabidopsis mutants were resistant to the biotrophic pathogen *Hyaloperonospora arabidopsidis*, whereas hypermethylated mutants were susceptible.

Numerous studies of plants’ defences have provided detailed information about how plants recognise attackers, the resulting modulation of hormonal pathways, and effects at multiple metabolic and physiological levels^34–36^. Diverse priming agents and biotic stresses have been applied to diverse plant taxa in various developmental stages. Clearly, it would be helpful to identify general patterns in responses, and their relationships (if any) with priming agents or antagonists. Therefore, we have sought such patterns and relationships through review of seed priming documented for various crops and meta-analyses of studies of priming of Arabidopsis plants. As effects of dosage and priming agent may be plant-species dependent, we focused our meta-analysis on one species and used Arabidopsis as a model. This is the first meta-analysis of defence priming in any plant species.

## Results

### Seed priming in Arabidopsis

Evidence of enhanced resistance to biotic stresses has been found in several plant systems after priming seeds’ defences using biological organisms or chemical biostimuli (see Table 1). Defence priming appears to generally enhance plants’ health, either by reducing effects of herbivore damage or disease symptoms, or by impairing the growth of populations or replication of pests or pathogens.

**Table 1:** Overview of seed priming studies.

Host Plant (HP)	Priming Stimulus*	Stress Agent (ASA)**	Trait	Priming***	Reference
*Lycopersicon esculentum Mill. cv. PKM-1*	*Bacillus subtilis (TN_Vel-35) (b)*	*Alternaria solani (f)*	**HP:** Gene expr. & enz. activity (*POX, PPO*)│Growth, yield, nutrient uptake, germination, vigour **ASA:** Disease symptoms	HP (+│+) ASA (−)	Babu *et al*. (2015)
*Lycopersicon esculentum Mill. cv. PKM-1*	*Azotobacter chroococcum (KR_Tri-17) (b)*	*Alternaria solani (f)*	**HP:** Enz. activity (*POX*, *PPO*)│Growth, yield, nutrient uptake, vigour **ASA:** Disease symptoms	HP (+│+) ASA (−)	Babu *et al*. (2015)
*Lycopersicon esculentum Mill. cv. PKM-1*	*Bacillus cereus KA_Mys-39 (b)*	*Alternaria solani (f)*	**HP:** Enz. activity (*POX, PPO*)│Growth, yield, nutrient uptake, germination, vigour **ASA:** Disease symptoms	HP (+│+) ASA (−)	Babu *et al*. (2015)
*Pisum sativum L*.	*Pseudomonas chlororaphis MA 342 (b)*	*Acyrthosiphon pisum (a)*	**ASA:** Population growth	ASA (−)	Hamada *et al*.^27^
*Capsicum annuum L. cv. Bukwang*	*Bacillus gaemokensis (PB69)* metabolites *(b)*	*Pseudomonas syringae pv. lachrymans (b) Xanthomonas axonopodis pv. vesicatoria (b)*	**HP:** Gene expr. (*LOX*)│Growth, yield **ASA:** Disease symptoms	HP (+│+) ASA (−)	Song *et al*.^18^
*Cucumis sativus L. cv. Backdadagi*	*Bacillus gaemokensis (PB69)* metabolites *(b)*	*Pseudomonas syringae pv. lachrymans (b) Spodoptera litura (l)*	**HP:** Gene expr. (*LOX*)│Growth, yield **ASA**: Disease symptoms, expansion rate, survival rate	HP (+│+) ASA (−)	Song *et al*.^18^
*Gossypium hirsutum*	*Beauveria bassiana (f)*	*Aphis gossypii (a)*	**ASA:** Population growth	ASA (−)	Castillo Lopez *et al*. (2014)
*Lycopersicon esculentum Mill. cv. Oogata-fukuju*	*Trichoderma harzianum (TriH_JSB27) (f)*	*Ralstonia solanacearum (b)*	**HP:** Gene expr. & enz. activity (PAL)│Growth, yield, nutrient uptake, germination, vigour **ASA:** Disease symptoms	HP (+│+) ASA (−)	Jogaiah *et al*.^15^
*Lycopersicon esculentum Mill. cv. Oogata-fukuju*	*Trichoderma harzianum (TriH_JSB36) (f)*	*Ralstonia solanacearum (b)*	**HP:** Growth, yield, nutrient uptake, germination, vigour **ASA:** Disease symptoms	HP (+) ASA (−)	Jogaiah *et al*.^15^
*Lycopersicon esculentum Mill. cv. Oogata-fukuju*	*Penicillium chrysogenum (PenC_JSB41) (f)*	*Ralstonia solanacearum (b)*	**HP:** Gene expr. & enz. activity (PAL)│Growth, yield, nutrient uptake, germination, vigour **ASA:** Disease symptoms	HP (+│+) ASA (−)	Jogaiah *et al*.^15^
*Lycopersicon esculentum Mill. cv. Oogata-fukuju*	*Phoma multirostrata (PhoM_JSB17) (f)*	*Ralstonia solanacearum (b)*	**HP:** Growth, yield, nutrient uptake, germination, vigour **ASA:** Disease symptoms	HP (+) ASA (−)	Jogaiah *et al*.^15^
*Helianthus annuus L. cv. Morden*	*Trichoderma harzianum PGPFYCM-14 (f)*	*Plasmopara halstedii (o)*	**HP:** Growth, yield, nutrient uptake, germination, vigour **ASA:** Disease symptoms	HP (+) ASA (-)	Nagaraju *et al*. (2012)
*Picea abies*	JA	*Hylobius abietis (bc)*	**HP:** Growth **ASA:** Attack, girdling	HP (0) ASA (−)	Berglund *et al*.^26^
*Solanum lycopersicum cv Carousel*	JA^a^	*Tetranychus urticae (s) Myzus persicae (a) Manduca sexta (l) Botrytis cinerea (f)*	**HP:** Gene expr. (*PinII*)│Growth, yield **ASA:** Disease symptoms, population growth, fecundity, survival	HP (+│0) ASA (−)	Worrall *et al*.^20^
*Capsicum annuum L. cv. Bukwang*	BTH^b^	*Pseudomonas syringae pv. lachrymans (b)Xanthomonas axonopodis pv. vesicatoria (b)*	**HP:** Gene exp. (*PR1*) **ASA:** Damage symptoms	HP (+) ASA (−)	Song *et al*.^18^
*Cucumis sativus L. cv. Backdadagi*	BTH^b^	*Pseudomonas syringae pv. lachrymans (b)*	**HP:** Gene expr. (*PR2*)│Growth, yield **ASA:** Disease symptoms, expansion rate	HP (+│0) ASA (−)	Song *et al*.^18^
*Solanum lycopersicum cv Carousel*	BABA^c^	*Oidium neolycopersici (f)*	**ASA:** Colonization	ASA (−)	Worrall *et al*.^20^
*Picea abies*	NIA (v:B3)	*Hylobius abietis (bc)*	**HP:** Growth **ASA:** Girdling	HP (0) ASA (−)	Berglund *et al*.^26^
*Picea abies*	NIC (v:B3)	*Hylobius abietis (bc)*	**HP:** Growth **ASA:** Attack, girdling	HP (0) ASA (−)	Berglund *et al*.^26^
*Hordeum vulgare L*.	Thiamine (v:B1)	*Rhopalosiphum padi (a) Sitobion avenae (a)*	**ASA:** Population growth, fecundity, settlement	ASA (−)	Hamada & Johnsson^66^
*Pisum sativum L*.	Thiamine (v:B1)	*Acyrthosiphon pisum (a)*	**ASA:** Population growth	ASA (−)	Hamada & Johnsson^66^
*Avena sativa L*.	Thiamine (v:B1)	*Rhopalosiphum padi (a)*	**ASA:** Population growth, fecundity, settlement	ASA (−)	Hamada *et al*.^27^
*Hordeum vulgare L*.	Thiamine (v:B1)	*Myzus persicae (a) Rhopalosiphum padi (a)*	**ASA:** Population growth, fecundity, settlement, lifespan	ASA (−)	Hamada *et al*.^27^
*Pisum sativum L*.	Thiamine (v:B1)	*Myzus persicae (a) Acyrthosiphon pisum (a)*	**ASA:** Population growth, settlement	ASA (−)	Hamada *et al*.^27^
*Triticum aestivum L*.	Thiamine (v:B1)	*Myzus persicae (a) Rhopalosiphum padi (a)*	**ASA:** Population growth, fecundity, settlement, lifespan	ASA (−)	Hamada *et al*.^27^
*Pennisetum glaucum (L.)*	Thiamine (v:B1)	*Sclerospora graminicola (o)*	**HP:** Enz. activity (*LOX*)│Growth **ASA:** Disease symptoms	HP (+│+) ASA (−)	Pushpalatha *et al*.^62^
*Pennisetum glaucum (L.)*	Riboflavin (v:B2)	*Sclerospora graminicola (o)*	**HP:** Growth, yield, germination, vigour **ASA:** Disease symptoms	HP (+) ASA (−)	Pushpalatha *et al*.^62^
*Pennisetum glaucum (L.)*	Niacin (v:B3)	*Sclerospora graminicola (o)*	**HP:** Growth, yield, germination, vigour **ASA:** Disease symptoms	HP (+) ASA (−)(−)	Pushpalatha *et al*.^62^
*Pennisetum glaucum (L.)*	MSB (v:K3)	*Sclerospora graminicola (o)*	**HP:** Growth, yield, germination, vigour **ASA:** Disease symptoms	HP (+) ASA (−)	Pushpalatha *et al*.^62^

**Priming Agents:** JA = Jasmonic acid, BABA = beta-aminobutyric acid, MSB = menadione sodium bisulphite, NIA = nicotinic acid, NIC = nicotinamide, BTH = benzothiadiazole, v: = vitamin:type. **Antagonist Stress Agent:** (s) = spider mite, (a) = aphid, (bc) = beetle, coleoptera, (l) = caterpillar, lepidopteran, (f) = fungus, (b) = bacteria, (o) = oomycota. **Response Trait:** trait used to assess priming effect: e.g. growth, damage symptoms or gene activity (PAL = Phenylalanine ammonia lyase, POX = peroxidase, PPO = polyphenol oxidase, LOX = lipoxygenase). **Priming:** Evidence of phenotypic differences between primed and un-primed plants, measuring directly on host plant (HP) or indirectly as antagonist stress agent response (ASA); (+) = enhanced, (−) reduced, (0) = no difference.

^a^JA had a negative effect on *Solanum lycopersicum* primary root length.

^b^BTH had a positive effect on *Spodoptera litura* weight in *Cucumis sativus* L. cv. Backdadagi, and a negative effect on *Capsicum annuum* L. cv. Bukwang shoot length.

^c^BABA had a positive effect on mean area of lesions caused by *Botrytis cinerea*.

More than 200 studies identified through the Web of Science searches investigated effects of priming seeds on the subsequent growth and development of Arabidopsis plants, but far fewer (11) reported effects of defence priming on biotic stress resistance, and none included the model plant Arabidopsis. With the diverse experimental backgrounds meta-analyses could not be performed on this dataset. However, a general conclusion is that priming with (for instance) vitamins and plant hormones has reportedly enhanced the resistance of many crops to a wide array of antagonists. In addition, increases in expression of defence genes recorded in the 11 studies were generally interpreted as increases in resistance, although neither general nor specific metabolomic responses were necessarily considered.

### Meta-analysis data

Of the 835 papers initially identified by Web of Science searches, 77 (describing 296 experiments) met the inclusion criteria. Of the excluded studies, 52% lacked information about the priming agent or biotic stress or the study was irrelevant, ca. 38% did not include experiments with wild-type *Arabidopsis thaliana* plants, ca. 3% were not peer-reviewed research articles, and 2% did not include data on performance of the antagonist. The remaining ca. 6% lacked information about sample sizes or errors, had poor figure resolution, included retracted or un-available parts or were studies of transgenerational priming.

The 296 studies (all included in the meta-analyses) documented results of applying defence priming treatments to foliage or root tissue, but not seed priming, except for a few studies of priming via soil enrichment by bacterial or fungal agents^37–40^.

### Plant priming increases resistance to biotic stress

**Figure 1:**
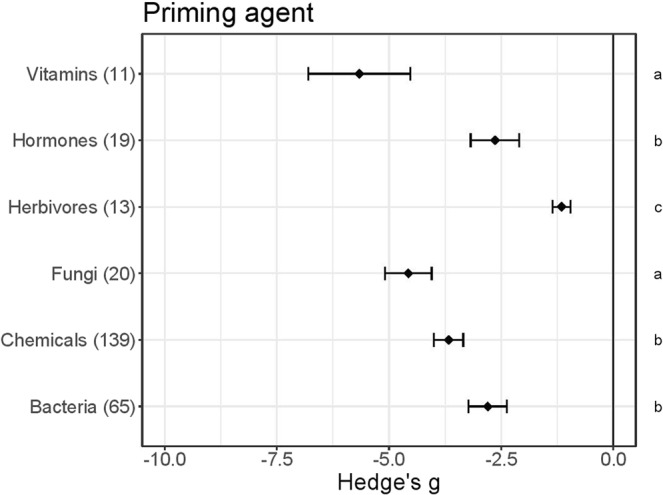
Resistance effects of priming Arabidopsis plants with indicated agents. Results of meta-analysis of data obtained from 267 experiments described in 77 publications. Negative values imply that primed plants were more resistant (less damaged or associated with lower pest fitness) than unprimed controls. Numbers of experiments are shown in brackets, and symbols specify means of Hedge’s g ± SE bars, equivalent to effects of groups of priming agents (Vitamins, Hormones, etc.). Different letters along the right-hand axis indicate significant differences according to the Kruskal Wallis test (α = 0.05) followed by Dunn’s post-hoc test to rank differences (α = 0.05).

To meet our objective of identifying patterns in priming responses we focused on experiments involving application of priming treatments to the model plant Arabidopsis (in vegetative stages), to exploit the large pool of relevant available information^41^. We used data drawn from 267 independent Arabidopsis defence priming experiments, reported in 77 papers, in our meta-analyses. In all of these experiments whole plants were exposed to either selected bio-stimuli or live organisms in the priming treatments. In almost all of the experiments priming enhanced resistance to biotic stress, on average (Fig. 1, Hedge’s g < 0). Ca. 7% (19 of 267 studies) suggested that it had no or negative effects on plant resistance (Hedge’s g > 0). Vitamins and microorganisms proved to be stronger primers, providing better general protection, than herbivores (Dunn’s test, P < 0.05). In addition, fungi primed Arabidopsis plants more strongly than bacteria (Dunn’s test, P = 0.002)). When ranking individual priming agents across all categories, priming with riboflavin and BABA yielded the highest improvement of plant resistance to biotic stresses, whereas aphids, caterpillars and ologigalacturonides (OGs) performed as the worst priming agents (Fig. S1).

### No general advantage of self-priming

**Figure 2:**
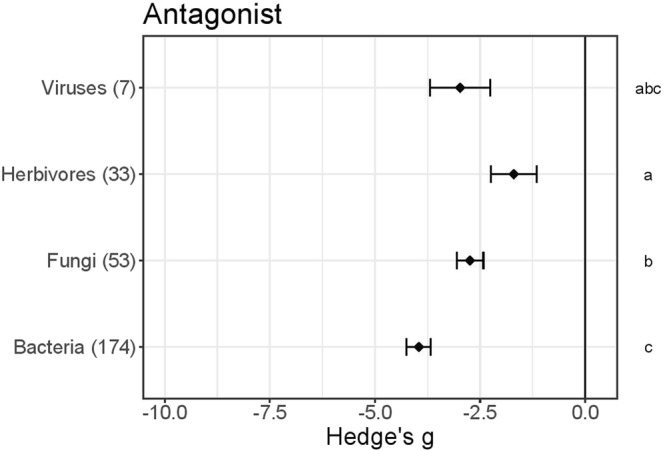
Enhancement of primed Arabidopsis plants’ resistance to indicated antagonists (i.e. ASA in Table 1). Results of meta-analysis of data obtained from 267 experiments described in 77 publications. Hedge’s g indicates the treatment effect for each taxonomic group of antagonists, and negative values imply that primed plants were less damaged (or hosted less fit antagonists) than unprimed controls. Numbers of relevant experiments are shown in brackets, and symbols specify means of Hedge’s g ± SE bars. Different letters along the right-hand axis indicate significant differences according to the Kruskal Wallis test (α = 0.05) followed by Dunn’s test post-hoc test to rank differences (α 0.05).

As several organisms had been used to prime the plants, we investigated their relative efficacy and dependence of their effectiveness on the antagonist used in the tests (Fig. 2). The meta-analyses suggested that priming with organisms is more likely to protect plants against bacterial and fungal antagonists than against herbivores (Dunn’s test, P < 0.05). In addition, there was no significant indication that “self” priming (i.e. priming by an organism that is later used as a stressor) was either more or less advantageous than priming by another organism. For example, Kruskal-Wallis rank sum tests detected no significant differences (P > 0.05) in effects of priming with fungal and non-fungal agents on fungal infections (N = 53), or effects of priming with herbivore and non-herbivore agents on herbivore damage (N = 33). However, bacterial “self” priming (Hedge’s g = −3.7 ± 0.6, N = 44) provided weaker protection than fungal priming against bacteria (Hedge’s g = −5.5 ± 0.7, N = 11, Dunn’s test, P < 0.05; Fig. S2). Note, throughout the paper, data in x ± y format are means ± standard errors.

### Evidence of organismal-specific priming effects

**Figure 3:**
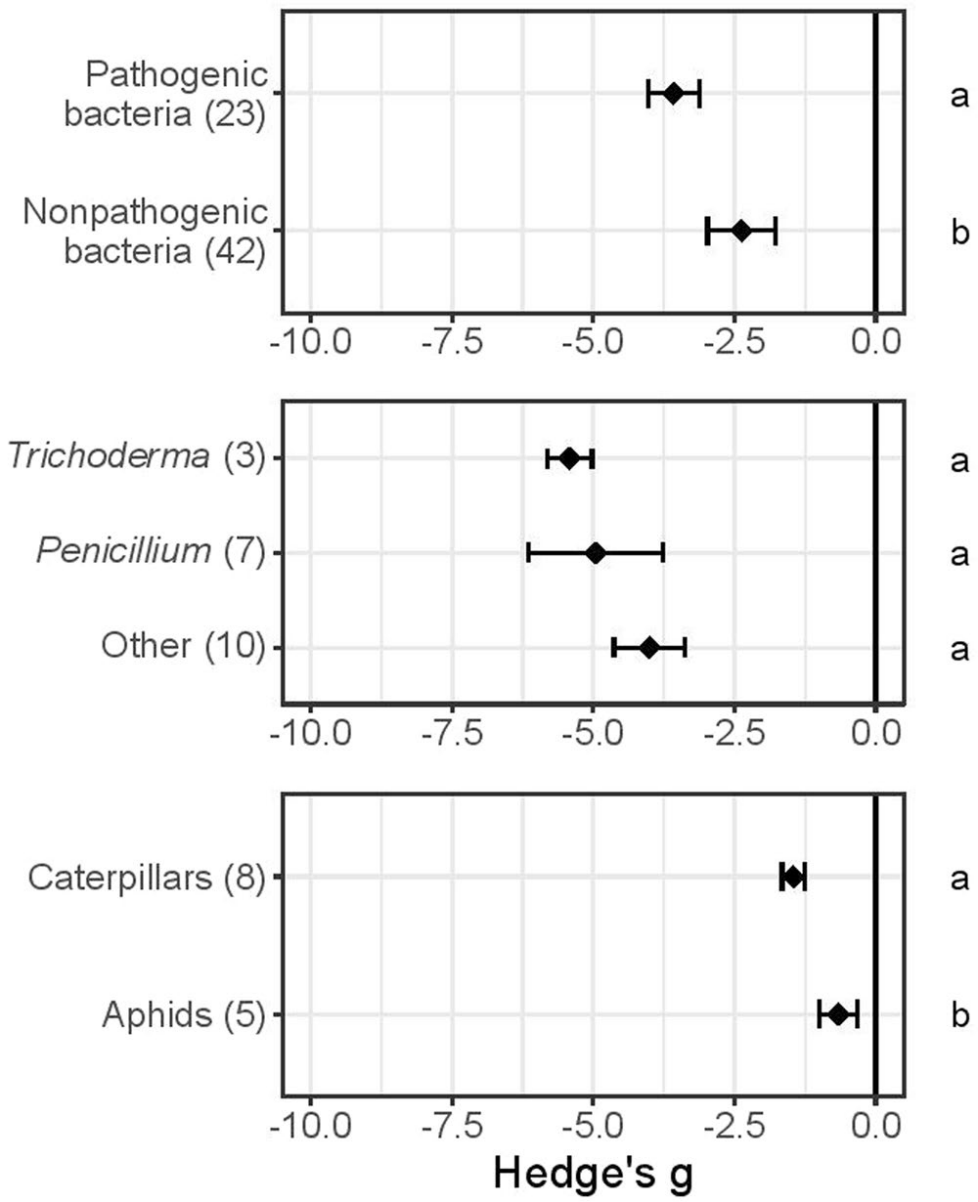
Enhancement of primed Arabidopsis plants’ effects on indicated sub-groups of groups of antagonists (ASA in Table 1). Negative mean values of Hedge’s g indicate that primed plants were less damaged (or hosted less fit antagonists) than unprimed controls. Results show: (**a**) differing effects of priming on pathogenic and non-pathogenic bacteria; (**b**) lack of significant differences in effects on fungal sub-groups (Penicillium, Trichoderma and “Other” (e.g. Phoma and Saccharomyces cerevisiae); (**c**) differing effects on aphid and caterpillar herbivores. Different letters along the right-hand axis indicate significant differences according to the Kruskal Wallis test, Wilcoxon rank sum test or Student’s t-test (α 0.05), followed by post-hoc Dunn’s test (α 0.05).

We also assessed variations in reported effects of defence priming among sub-groups of organisms (Fig. 3). Pathogenic bacteria reportedly induced significantly stronger resistance (Hedge’s g = −3.6 ± 0.5, N = 23) than non-pathogenic bacteria (Hedge’s g = −2.4 ± 0.6, N = 42; Wilcoxon rank sum test, P < 0.001; Fig. 3a). This result appears, however, to be highly affected by the difference in antagonist composition between the groups. Herbivores for example make up ca. 43% of the antagonists in the non-pathogenic bacteria dataset whereas pathogenic bacteria have 9% herbivores. When excluding herbivores, non-pathogenic bacteria appears to induce slightly stronger resistance (Hedge’s g = −3.7 ± 1.0, N = 24) compared to pathogenic bacteria (Hedge’s g = −3.6 ± 0.5, N = 21; Wilcoxon rank sum test, P = 0.04). In addition, as shown in Fig. 3b, *Trichoderma* induced somewhat higher resistance (Hedge’s g = −5.4 ± 0.4, N = 3) than *Penicillium* (Hedge’s g = −5.0 ± 1.2, N = 7), which induced stronger priming than “Other” fungi (including *Phoma* sp. and baker’s yeast; Hedge’s g = −4.0 ± 0.6, N = 10). However, these differences were not significant according to the Kruskal-Wallis rank sum test, possibly because the sample numbers did not provide sufficient statistical power. Herbivores used as priming agents were divided into two classes: aphids (Hedge’s g = −0.7 ± 0.3, N = 5) and caterpillars (Hedge’s g = −1.5 ± 0.2, N = 8). Caterpillars had significantly stronger priming effects than aphids according to Student’s t-test (P = 0.048, Fig. 3c).

### Evidence of priming effects of plant-related elicitors

**Figure 4:**
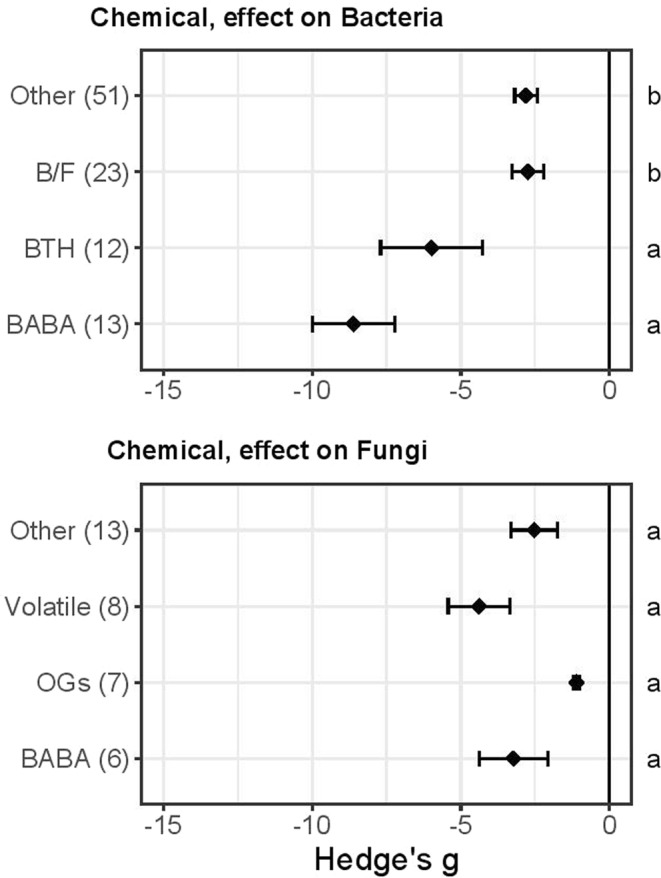
Differences in effects of priming by chemicals on bacterial and fungal antagonists. (**a**) Effects on bacteria of beta-aminobutyric acid (BABA), benzothiadiazole (BTH), compounds derived from bacteria or fungi (B/F), and associated compounds (“Other”). B/F included flg22, lipopolysaccharides, hairpin protein, ergosterol, siderophores, cyclic dipeptides, and a bacterial quorum-sensing molecule. Data from experiments with chemicals used in ≤2 studies were pooled, forming the category “Other”. These include pentanol, dehydroabietinal, steroid, oligogalacturonides, 1,2-benzisothiazol-3(2 H)-one1,1-dioxide (BIT), azelaic acid, E-2-hexenal, glutathione, glutathione disulphide, pipecolic acid, sulphanilamides, amino acids (Gly, Cys, Ser, Ala, Asp, Asn, Glu), and compounds derived from algae or oomycota. (**b**) Effects on fungi of volatiles, oligogalacturonides (OGs), BABA and other chemicals. In this case data from experiments with chemicals described in only one article were pooled, and they include thymol, allose, glycine, abietic acid, 2,6-dichloroisonicotinic acid, galacturonic acid, indole-3–carboxylic acid, hypoxanthine, hexanoic acid, BTH, and flg22. Symbols specify mean values of Hedge’s g ± SE. Negative values imply that primed plants were more resistant (less damaged or associated with lower pest fitness) than unprimed control plants. Different letters along the right-hand axis indicate significant differences according to the Kruskal Wallis test and comparisons performed with post-hoc Dunn’s test (α 0.05).

Chemical priming was included in many experiments (N = 139) and effects of diverse biotic stressors were tested. The tested chemical compounds included two classes of phytohormones, jasmonic acid compounds (JA and MeJA; Hedge’s g = −2.8 ± 1.1, N = 9) and salicylic acids (Hedge’s g = −2.5 ± 0.4, N = 10), which did not apparently differ in priming strength (Wilcoxon rank sum, P > 0.05). There was also no significant difference between effects of the B vitamins thiamine (Hedge’s g = −4.7 ± 1.5, N = 7) and riboflavin (Hedge’s g = −7.4 ± 1.6, N = 4; Wilcoxon rank sum, P > 0.05). To further investigate priming compounds’ effects, we tested differences in priming effects of four kinds of elicitors —BABA, BTH, selected bacterial and fungal compounds (B/F), and “Other” — on bacterial antagonists. The results indicated that, generally, chemical priming protects Arabidopsis against bacterial antagonists (Fig. 4a). Moreover, BABA provides the strongest protection (Hedge’s g = −8.6 ± 1.4, N = 13), followed by BTH (Hedge’s g = −6.0 ± 1.7, N = 12), “Other” (Hedge’s g = −2.9 ± 0.4, N = 50), and B/F (Hedge’s g = −2.7 ± 0.5, N = 23). Differences between chemical priming agents on bacterial antagonists were confirmed statistically (Dunn’s test, P < 0.05). In addition, several chemicals reportedly primed Arabidopsis against fungal infections (Fig. 4b), including BABA (Hedge’s g = −3.2 ± 1.2, N = 6), volatiles (Hedge’s g = −4.4 ± 1.0, N = 8), oligogalacturonides (OGs, Hedge’s g = −1.1 ± 0.1, N = 7) and “Other” compounds (Hedge’s g = −2.5 ± 0.8, N = 13). However, we found no significant differences between effects of those compounds (Kruskal-Wallis rank sum, P > 0.05).

### Quality control of the database

Publication biases were evaluated with funnel plots, which were either symmetric suggesting that data were unbiased (for example, for herbivore priming effects and resistance), or slightly asymmetric, suggesting that data were slightly biased (Fig. S3). However, Fail-safe numbers indicating the number of non-significant findings required to reject the outcome of a meta-analysis suggested that our database was highly representative (Table S4).

## Discussion

### Fungi and vitamins prime Arabidopsis plants’ defences most strongly

There is ample evidence that seed defence priming with diverse agents can protect various plant species against diverse antagonists (Table 1). Curiously, we found no published studies that assessed effects of priming defences of Arabidopsis seeds, apart a few indicating that enrichment of soil by bacterial or fungal agents may have indirect priming effects^40,42–44^. Regarding priming at the plant stage, we found 835 Arabidopsis studies of which 77, describing 267 experiments, fulfilled our requirements. More specifically, they provide information on effects of priming by organisms or plant-derived elicitors on defences against bio-stresses, including quantifiable details about antagonist responses (Supplementary Material List S5). Meta-analysis of this information confirmed that such priming generally enhances pest or pathogen resistance in Arabidopsis, and that fungi and vitamins have stronger effects than other tested agents, inducing stronger resistance to bacterial and fungal antagonists than to herbivores. The meta-analyses summarize indications of priming strength and antagonist specificity in previous studies, which may support future efforts to study and understand defence priming in Arabidopsis and other plants.

### Priming agents and their generality

#### Organismal priming: microorganisms

An increasing number of studies suggest that plant resistance may be orchestrated interactively with associated organisms^45^ and those interactions could potentially be commercially manipulated^46^. Below-ground relationships with mycorrhizal fungi and bacteria, for example, not only promote growth, but also reduce damage to above-ground parts^47^, pre-inoculation of fungal endophytes modulates later plant disease development^47–49^, and spread of *Heterobasidion* (stem rot) to healthy spruce trees may be avoided by applying *Phlebiopsis gigantean* fungal spores to stumps^50^. Spontaneous associations between microbiomes and plants further suggest that cross-kingdom associations may provide overlooked promotion of plant growth and development^16,17^. Generally positive effects of priming by bacteria and fungi have been reported in Arabidopsis (Figs 1–4), confirming the potential to use microorganisms commercially for priming plants’ defences, as knowledge of the specificity and strength of various organisms’ effects increases.

Our meta-analyses suggest that defence priming by certain groups of organisms may provide better general protection than others. Fungi appear as the stronger of the tested organismal priming agents in Arabidopsis (Figs 1 and 3b), and bacteria follow closely (Fig. 3a). Organismal priming may offer some advantages over use of chemicals. For example, cold season tall fescue grasses that host defensive endophytic symbionts (*Neotyphodium*) have selective advantages in the presence of herbivores^51^, illustrating the potential importance of associated organisms for mutual protection. There is increasing awareness that the microbiota associated with plants is constantly filtered by the taxa, organs, and developing tissues present^16^. Moreover, plants and associated organisms share both semiochemical signals^17,19,52^ and evolutionary histories^53–55^. Unravelling such relationships and exploiting them for plant production may offer environmentally sound agricultural strategies. The diversity of natural interactions and associations is vast but increasing knowledge of the patterns involved is enhancing our ability to recognize and apply them^8,10,56^.

#### Organismal priming: Herbivores

As evidenced by meta-analyses, herbivores appear as generally weak priming agents (Figs 1 and 3c). Higher organisms induce defences in plants, as they give rise to immediate activation of defensive pathways and upregulated pools of specialised defence products^57,58^. Long-lasting trans-generational resistance shaped by herbivory has also been documented in Arabidopsis and tomato by Rasmann, *et al*.^12^. Our meta-analyses indicate that herbivores are generally weak priming agents (Figs 1 and 3c), although they induce immediate activation of defensive pathways and upregulation of pools of specialised defence products^57,58^. Internal herbivore feeders (e.g. miners and gallers) have more intimate associations with their hosts, but maintaining them in culture for experimental purposes is demanding, which may explain the scarcity of mechanistic and detailed studies of their impact on defence priming in the literature.

In addition, any reported priming of Arabidopsis plants included in our meta-analyses had weak anti-herbivory effects. In contrast, several studies found that seed priming effectively prevented herbivory in various crop systems (Table 1 and references herein, also see Rasmann, *et al*.^12^). Thus, we cannot exclude the possibility that the success of priming treatments against herbivore attacks may depend on the developmental stage at which they are applied to plants.

#### Non-organismal chemical priming: Vitamins

Vitamins are strong chemical priming agents according to our meta-analyses (Fig. 1). Moreover, they are essential, natural organic substances and many have additional defensive functions *in planta*. Hence, they have commercially appealing properties for priming purposes, including non-phytotoxicity and potentially growth-stimulating properties (Table 1), and B vitamins in particular are widely used as priming agents^59–62^.

Thiamine (vitamin B_1_) is an antioxidant produced by plants, bacteria, and fungi^60,63^. It naturally functions as a coenzyme in several metabolic pathways, including the Krebs cycle, glycolysis, and pentose phosphate pathway^63,64^. In Arabidopsis, Tunc-Ozdemir, *et al*.^64^ found that various stress treatments (cold, salt and paraquat) led to accumulation of thiamine, accompanied following paraquat treatment with reduced production of reactive oxygen species (ROS). Observations of enhanced defence responses to infection by *Pseudomonas syringa*e pv *tomato* bacteria in various plants following foliar applications of thiamine confirmed that it has diverse, species- and genotype-specific priming effects^24,65^. When applied to seeds, thiamine also enhances resistance of several crops (including pea, barley, oat, wheat, and millet) against aphid pests^27,66^ and fungal infections^62^.

Vitamin-B_2_ (riboflavin) is also produced naturally in plants, but its effects as a priming agent have not been frequently tested. However, Azami-Sardooei, *et al*.^67^ reported that priming with B_2_ enhanced resistance to the pathogen *B. cinerea* in bean but not tomato plants. The authors cited argue that the latter may have been insensitive to B_2_ priming because the tomato plants already had sufficient endogenous levels of B_2_, lacked riboflavin receptors, or were unable to absorb it adequately.

Vitamin B_3_ is another natural metabolite in plants, niacin, that has frequently been used to prime plants. More strictly, niacin refers to nicotinamide and the closely related nicotinic acid (also with B_3_ activity). Nicotinamide is released by the enzyme poly (ADP-ribose) polymerase (PARP) in response to oxidative stress causing single-strand breaks in DNA^68,69^. Thus, as it is formed during oxidative stress, it putatively functions as a general signal and stress response^59^. Plants primed with B_3_, in either nicotinic acid or nicotinamide forms^70^, reportedly have enhanced resistance to both subsequent biotic^26,61^ and abiotic^26,71^ stressors.

Vitamins B_1_ and B_3_ have also been used as priming agents for a range of crops (including barley, wheat, and pea) without causing detectable phenotypic changes. Hamada, *et al*.^27^ and Hamada and Jonsson^66^ found that vitamin B_1_ protected several crops against aphid attacks, while Berglund, *et al*.^26^ found that B_3_ protected spruce against pine weevils. The lack of detected phenotypic responses to treatment with these agents is very promising as the ideal defence priming agent would have no or minimal performance penalties for the host, compared to unprimed controls^22^. In our meta-analyses there were too few studies to distinguish between effects of specific vitamins. However, our results confirm that vitamins may be attractive agents for priming plants of various species, applied either in vegetative stages (as in Arabidopsis studies) or seeds (as in studies with assorted non-model crop species).

#### Non-organismal chemical priming: Defence elicitors

Another potentially strong priming agent is the signalling compound aminobutyric acid isomer BABA (Fig. 4a)^20^. This is usually present at levels so low that it was only recently proven to be synthesised in highly diverse plant species in response to several kinds of pathogens^72^. Our meta-analyses show that BABA-priming of Arabidopsis plants has provided strong protection against bacterial diseases and, to a lesser degree, fungal diseases (Fig. 4a,b). Wilkinson, *et al*.^73^ also detected strong long-lasting effects of BABA against *Botrytis cinerea* post-harvest infections in tomatoes with no yield penalty. Elucidation of mechanisms behind these diverse effects may provide highly interesting insights and opportunities.

In contrast to BABA, oligogalacturonides (OGs) appear to have weak priming efficiency against fungal diseases (Fig. 4b). OGs comprise a diverse group of defence signalling molecules that are degradation products of pectin in plants’ cell walls. When disrupted they break into fragments, or so-called Damage Associated Molecular Patterns (DAMPs) that induce plant immune responses via an MPK-dependent pathway^74–76^. Thus, the finding that OGs did not score highly as priming agents in our analyses was surprising, but may be due to their diversity, and OG specificity may warrant further attention in future studies. Only general effects of OGs have been tested in seven Arabidopsis studies as yet, so it is too early to draw robust conclusions about their potential utility as priming agents.

Priming of plants with jasmonates is highly efficient, and even deters relatively large insects such as pine weevils from de-barking coniferous plants^26^. Martinez-Medina *et al*.^22^ argue that priming may come at an initial cost, that is outweighed by later benefits, but methyl-jasmonate  (MeJA) appears to come with fairly high cost because it stunts growth, and causes plants to reallocate resources towards specialized metabolism e.g. terpenoid production in spruce^77^. Li, *et al*.^78^ found that MeJA applications may also cause changes in cuticle thickness and composition, and increases in densities of trichomes and stomata, as well as reductions in height and biomass, in various species (e.g., tomato, sunflower and soybean). Thus, although application of MeJA efficiently enhances herbivore resistance its value as a priming agent is questionable due to its undesirable phenotypic effects.

Previous meta-analyses have shown that green leaf volatiles (GLV) may protect plants against bacterial, fungal and herbivorous antagonists^57,79^. Despite the evidence of shared cross-kingdom chemical signalling, volatiles (and compounds derived from bacteria and fungi) did not collectively score high as priming agents in our meta-analyses. However, they are also highly heterogenous groups of compounds. Diverse expression-level responses to these compounds have been examined, and they have usually included responses of defence-associated pathways (for example JA or SA pathways) or pathways leading to specialised defence compounds (e.g., phenylpropanoids or glucosinolates, Table 1). Organisms secrete chemicals. Bacteria, for example, produce signaling molecules to regulate transcription and cell population density^80^ that may affect plant performance. Therefore, the mechanisms behind any organismal or chemical treatments may have shared mechanisms, which motivated a general illustration of efficiency order of all biostimulants included in this study (Fig. S1).

The increasing abundance of information about plants’ responses to stress conditions is rapidly increasing knowledge of the sophisticated metabolic and signalling networks involved in finetuning their defence responses. Understanding these responses and plants’ interactions with associated organisms may undoubtedly facilitate discovery of alternative strategies to protect plants, as noted by various authors^10,16,17,53,81^. However, the mechanisms involved in diverse responses must be elucidated to enable any generalisations.

### Potential bias and sources of error

The quality of the database used in any meta-analysis is a major concern, mainly because publications on any topic may be subject to biases that exaggerate evidence, as significant effects or relationships are more attractive for publication than non-significant effects or relationships^82^. In our case, a risk of overestimating effects was weakly suggested in the funnel plots (Fig. S3). However, that concern was strongly contradicted by Fail-safe numbers (Table S4), which indicate the number of conflicting studies needed to reject findings of a meta-analysis.

Another potential source of error in meta-analyses lies in the way parameters are chosen. There are endless ways to measure priming effects, due to the diversity of both potential experimental systems (e.g. between non-pathogenic and pathogenic bacteria) and biological settings of priming treatments. There is also often no general agreement between responses that may be dynamic, non-linear and dependent on both spatial and temporal parameters (e.g. when evaluating relationships between gene expression, enzyme activity, and metabolomic changes). Currently we have no general mechanistic understanding of priming, although potential MPK accumulation and epigenetic alterations have been suggested. Any measurable general mechanistic understanding of plant memory would be a huge step forward in assessing and documenting plant priming.

### Arabidopsis and general insights

The meta-analyses presented here are based on studies of the model species *Arabidopsis thaliana*. This is advantageous as it avoids potentially confounding variation in responses due to variations in the host plant, thereby assisting inter-study comparisons. The Col-0 accession, the first model plant to be sequenced was used in more than 85% of the studies included in our database and meta-analyses. Thus, the findings may not be relevant for all plant systems, but Arabidopsis provides a convenient model of higher plants generally, and the *Brassicaceae* specifically, offering high potential for molecular follow-up studies to unravel mechanisms underlying defence priming^41^.

Curiously, although diverse plant systems may be primed at the seed stage (Table 1), we identified no studies of defence  priming Arabidopsis seeds, so we could only consider aspects of priming this species at the plant stage. Moreover, we identified relatively few studies on priming of seeds of other species, although priming at the seed stage appears to be commercially attractive. This could be due to biased knowledge in this field of research, which is often driven by private seed companies^83^, so knowledge of seed-priming mechanisms could be protected by commercial interests.

### Future challenges

Despite advances in knowledge of priming, several challenges must be addressed before priming may be commercially viable^84^. Reliable priming methods must be established with detailed knowledge about priming strength for relevant crop species, and detailed information about priming stability and reliability will be expected by the customers. Successful priming increases plants’ overall performance under stress^21,22,85^, and to evaluate priming strength in any experimental test, it is desirable to include both physiological costs and performance benefits. This is not straightforward. Positive effects on yield and negative effects on an antagonist are convincing indications of successful priming, but bioassays are costly and elaborate to perform, and demanding to standardize^86^. A mechanistic understanding of priming might indicate convenient and cost-effective ways to assess priming. Assessments of MPK accumulation and epigenetic responses (DNA methylation/demethylation and histone modifications) are promising possibilities. However, they cannot stand alone and must be calibrated according to plant and antagonist performance.

**Figure 5:**
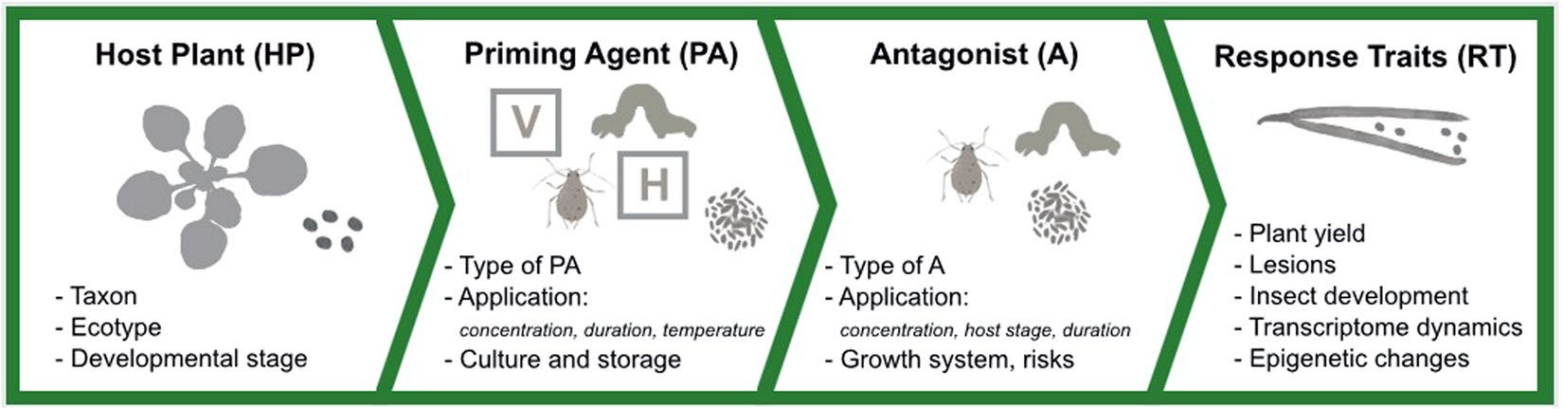
A hierarchical overview of relevant process elements (referred to in this paper) in the development of chemical and biological seed priming routines. Priming with live organisms involves three-way interactions, and optimisation of screening conditions for both host plant and priming agent may be required. Measured response traits may include variables indicating changes in host performance, symptoms or antagonist performance. Some response traits (e.g. molecular level changes) will require a priori calibration for correct interpretation in terms of costs or benefits for the plant. (Letters in squares refer to vitamins “V” and hormones “H”).

A priming agent can be applied in diverse ways, e.g. by spraying or submerging seeds, foliage or roots. Seeds have obvious advantages, as they can be treated evenly and precisely with little if any environmental impact or non-target ramifications^87^. Moreover, in contrast to plant priming, seed priming will also protect plants from the earliest stages of germination and throughout their development. Thus, seed treatment promises to be less labour intensive and more cost-efficient than treatment of plants^88^. However, the development of robust seed priming procedures will involve several optimization steps, including choices of plant system and priming agents, test conditions, stresses, and responses of both plants and antagonists (Fig. 5).

Ugena *et al*.^89^ developed a multi-trait high throughput screening method of biostimulants on Arabidopsis seeds to assess effects on growth and germination in response to subsequent salt stress. This work provides a rare resource for characterization of biostimulants towards plant health promotion.

## Conclusion

There is an urgent need for sustainable crop protection techniques and seed defence priming appears to be an attractive strategy. However, before it can be commercially applied several aspects, starting with the priming mechanism(s), must be elucidated. Assessments of priming treatments’ persistence (duration of priming effects) and the range of biotic stresses they may protect against are also warranted. The meta-analyses presented here convincingly show that priming has general potential for raising crop productivity. This is increasingly important as our agricultural systems are facing severe challenges including increasing demands, higher costs, and a changing climate.

## Material and Methods

### Literature search

Literature covering priming of seeds to enhance germination and growth extends back to the 1970s, but relatively few published studies have focused on the priming of seeds’ defences against biotic stress. Strategic searches of databases like Web-of Science detected few relevant papers, which address diverse systems. A much larger body of literature covers defence priming in the plant stage, and we used Web-of-Science to compile a database of these studies in *Arabidopsis thaliana* (hereafter Arabidopsis) that provides a model for studies of plant biology, and is a suitable study system to understand mechanistic responses in plants at the molecular scale^41^.

### Data selection

#### Seed defence priming

As already mentioned, Web of Science searches identified relatively few studies on defence priming in seeds. For example, a search for papers on the Topics seed priming *AND* biotic stress on 27 July 2018 resulted in 31 papers, published from 2009–2018 including 20 published in 2012–2018 that addressed seed priming. Of the latter, 15 covered enhancement of resistance to abiotic stress and just four documented defence priming against pathogens. Host plants considered in those 20 studies were mainly crops such as rice, barley, wheat, maize, tomato, onion, corn and *Brassica* species, and only four included defence priming with use of a specific compound (beta-aminobutyric acid, BABA) or pathogen (*Trichoderma* or a hemibiotrophic pathogen). The search also listed a few reviews on, for example, uses of macro-algal compounds as agricultural bio-stimulants^90^. Back-tracing through such reviews, as described for example by^18,60^, was a more rewarding search strategy, resulting in identification of most of the studies on defence priming in seeds listed in Table 1 (see List S6 for references).

#### Plant defence priming

To cover defence priming of Arabidopsis plants, we searched the Web of Science for documents of Article type in the Category Plant Sciences on the Topics Arabidopsis *AND* priming. This resulted in 509 hits (collected on the 17th and 18th of April 2018). An additional search for Articles in the Category Plant Sciences with Arabidopsis in the title on the Topics treatm* *AND* resistance *AND* plant defen?e resulted in 326 more hits (collected on the 2nd and 3rd of May 2018). Thus, the resulting database included 835 articles.

Papers that mentioned Arabidopsis as a main study organism in the title, abstract or author keywords were then selected. Fifty of the discarded papers were randomly chosen for quality checking, and scrutinised to verify that they did not include data on responses of Arabidopsis that should have been included according to the data selection criteria. Thus, although a few studies may have been overlooked by chance, we judge that the database is rich enough to fulfil the purpose of this study.

Next, in order to extract usable information from the identified papers, each reported study was examined and retained if:

1. It included experiments with a priming agent and a wild-type population of any specified ecotype of *Arabidopsis thaliana*. Priming and tests with any kinds of organs or tissues (e.g. seeds, plants, or roots) were allowed.
2. The priming agent was organismal (e.g., a herbivore, bacterium, fungus, or pathogen effector), or plant-related elicitor, e.g., beta aminobutyric acid (BABA), or flagellin (flg22), a plant hormone related to biotic stress, e.g., jasmonic acid (JA, or its methyl derivative, MeJA) or salicylic acid (SA), or any vitamin.
3. Experiments were detailed and information about the priming agent (its kind, concentration, treatment duration etc) was included.
4. Responses to any kinds of biotic stresses (e.g. any herbivore, bacterium, fungus or virus) were detailed, but not responses to un-groupable stress agents (e.g. singletons, oomycota or transgenic stresses).
5. The study provided information about both primed and unprimed plants, statistical data about their responses (averages and standard deviations, standard errors or confidence intervals), and sample sizes, as well as resistance effects in terms of quantified pest/pathogen performance. Experiments that solely quantified Arabidopsis defence responses (e.g. gene expression, metabolite accumulation, etc.) were excluded. Papers indicating pathogen performance based solely on use of RT-PCR and ELISA were also excluded (ca. eight papers were excluded on these grounds). Studies providing information on behavioural responses of herbivores, for example, data on feeding responses of aphids obtained using electrical penetration graphs showing durations and frequencies of phloem phase were included.

Experiments were *not* included if:

1. The authors had not specified treatments of the primed or control plants (regarding, for example, concentrations/amounts of primer used), or information about types of errors was incomplete.
2. Effects of an abiotic stress such as salt stress or drought, or the highly specific agents ethylene, abscisic acid (ABA), coroatine, catechol, silicon, quinolinate, mycotoxins, transgenic bacterial strains, and volatiles or exudates produced by transgenic plants were defined or regarded as priming agents.
3. Priming treatments included use of NADPH oxidase inhibitors (e.g. DPI), H_2_O_2_ scavengers (e.g. catalase), a mETC uncoupler (e.g. antimycin or rotenone), nitric oxide inhibitors (e.g. cPTIO, L-NAME and OA) or BABA-inhibitors (e.g. L-Glutamine).
4. Several priming agents, for instance two compounds, were combined.
5. They were intended to determine active components or sizes of the priming agent (e.g. via use of proteases or deacetylases).
6. Transgenerational priming was tested.

### Data extraction

Mean values and variances of resistance data were extracted from tables and figures in the remaining studies and listed according to priming agent and antagonist. The plugin “Figure Calibration” in ImageJ, available at: http://www.astro.physik.uni-goettingen.de/hessman/ImageJ/, was used to obtain data from plots when numbers were not available. If sample sizes were given as a range (e.g. 20–25 replicates), the lowest number was listed. If the resolution of a figure was too low to extract data, the study was excluded.

If a primer’s effects at several concentrations and/or time points were tested in an experiment, data pertaining to the strongest effect (positive or negative) were included. Bacterial growth assays presented at log-scale were log-transformed after data extraction. Repeated experiments were included if they were independent of each other. Effect size (Hedge’s g) was calculated using Rstudio as described by Del Re^91^.

If several parameters (e.g. bacterial growth and disease severity) of individuals were measured, the effect sizes obtained were aggregated into a single effect size according to the “BHHR” procedure^91^. After aggregation, 267 experiments described in 77 papers remained and were included in the database, and subsequent meta-analyses. Responses quantified in these experiments included lesion area, feeding damage, bacterial growth, infection, disease severity, spore production, feeding duration, population increase, herbivore weight, time to pupation and/or reproduction rate. The studies reporting these experiments are listed in the Supplementary Information (List S5). Funnel plots and Fail-safe numbers, indicating how many conflicting studies would be needed to reject the outcome of a meta-analysis, are also reported in the Supplementary Information (Fig. S3 and Table S4) in agreement with use of Funnel plots and Fail-safe numbers^92,93^.

After calculating the effect sizes, the experiments were divided into classes depending on the type of priming (Bacteria, Fungi, Herbivores, Chemicals, Hormones or Vitamins, with further division into sub-classes such as JA, SA, or B1) and biotic stressor (bacterial, fungal, herbivore, or viral) applied.

### Statistical analyses

QQ-plot and Shapiro tests were used to assess the normality of data distributions. The variance of the data was assessed with F- or Levene’s tests. When comparing two groups, statistical analyses were performed using the parametric Student’s t-test (with equal or unequal variance settings depending on the outcome from the variance test) and nonparametric Wilcoxon rank sum and Kolmogorov-Smirnov tests (for datasets with sufficiently equal and unequal variance, respectively). If more than two groups were compared, statistical analysis were performed using One-way ANOVA for normally distributed data and Kruskal Wallis tests for non-normally distributed data. Pairwise comparison after the Kruskal Wallis test was performed using post-hoc Dunn’s tests (with Benjamini-Hochberg methodology for P-value adjustment). A significance threshold of 0.05 was applied in all tests. R version 3.4.2 was used for all analyses and generating all plots.

Ethical and third parts issues do not apply to this submission as no experiments were performed and no copying of any material included.

## Supplementary information

Supplemental material

online data

## Data Availability

The data of this study is based on extracts from published papers available through scientific citation data bases, and it is available as open access on-line data https://springernature.figshare.com/s/19070f9acfb5182cce0b. Lists of references that were included to compile the data base are available in the up-loaded supplemental material document included in the submission.
