# Supplementary material for: Defence priming in Arabidopsis – a Meta-Analysis
Source: Sci Rep. 2019 Sep 16;9:13309. doi: 10.1038/s41598-019-49811-9 (PMC6746867; doi:10.1038/s41598-019-49811-9)
Supplement: Supplementary file 1 — Supplemental material [file 41598_2019_49811_MOESM1_ESM.pdf]

Supplementary material to:

Defence priming in Arabidopsis – a Meta-Analysis

Westman, Sara[1]; Kloth, Karen J.[1,2]; Hanson, Johannes[1]; Ohlsson, Anna B.[3]; Albrectsen, Benedicte R. [1,*]

Addresses:

[1] Umeå Plant Science Centre, Department of Plant Physiology, Umeå University, Umeå Plant Science Centre, Umeå, Sweden

[2] Laboratory of Entomology, Wageningen University, P.O. Box 16, 6700 AA Wageningen, The Netherlands

[3] Department of Industrial Biotechnology, School of Engineering Sciences in Chemistry, Biotechnology and Health (CBH), KTH Royal Institute of Technology, Stockholm, Sweden

* Corresponding author, Benedicte Riber Albrectsen, Benedicte.albrectsen@umu.se

Figure S1 Priming agent Efficiency order

Figure S2 Self-priming Hedge's g

Figure S3 Funnel plots

Tabel S4 Fail-safe numbers

List S5 reference list Meta-analysis

List S6 added references to Table 1

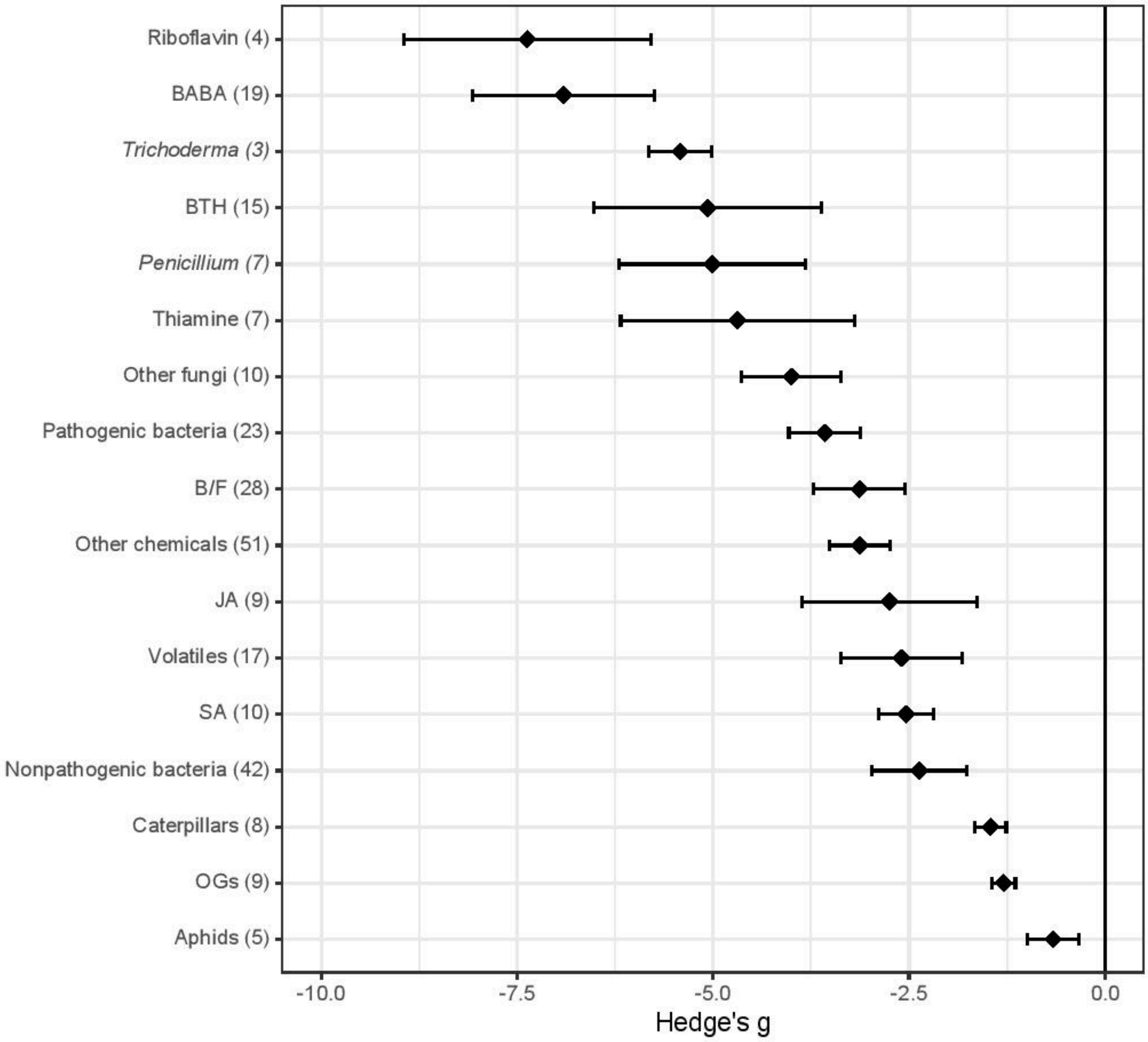

S1. Priming agent efficiency order. Priming agents are ranked on the y axis according to their effect on plant resistance to biotic stress. Results of meta-analysis of data obtained from 267 experiments described in 77 publications. A negative mean value of Hedge's g indicates that primed plants were less damaged (or hosted less fit antagonists) than unprimed plants. "Other fungi" include Phoma and Saccharomyces cerevisiae. Bacterial and fungi derived compounds (B/F) include flg22, lipopolysaccharides, hairpin protein, ergosterol, siderophores, cyclic dipeptides, and a bacterial quorum-sensing molecule. "Other chemicals" include dehydroabietinal, a steroid, 1,2-benzisothiazol-3(2H)-one1,1-dioxide (BIT), azelaic acid, glutathione, glutathione disulphide, pipecolic acid, sulphanilamides, amino acids (Gly, Cys, Ser, Ala, Asp, Asn, Glu), thymol, allose, glycine, abietic acid, 2,6-dichloroisonicotinic acid, galacturonic acid, indole-3–carboxylic acid, hypoxanthine, hexanoic acid, and compounds derived from algae or oomycote. Symbols specify mean values of Hedge's g ± SE. Sample size in brackets.

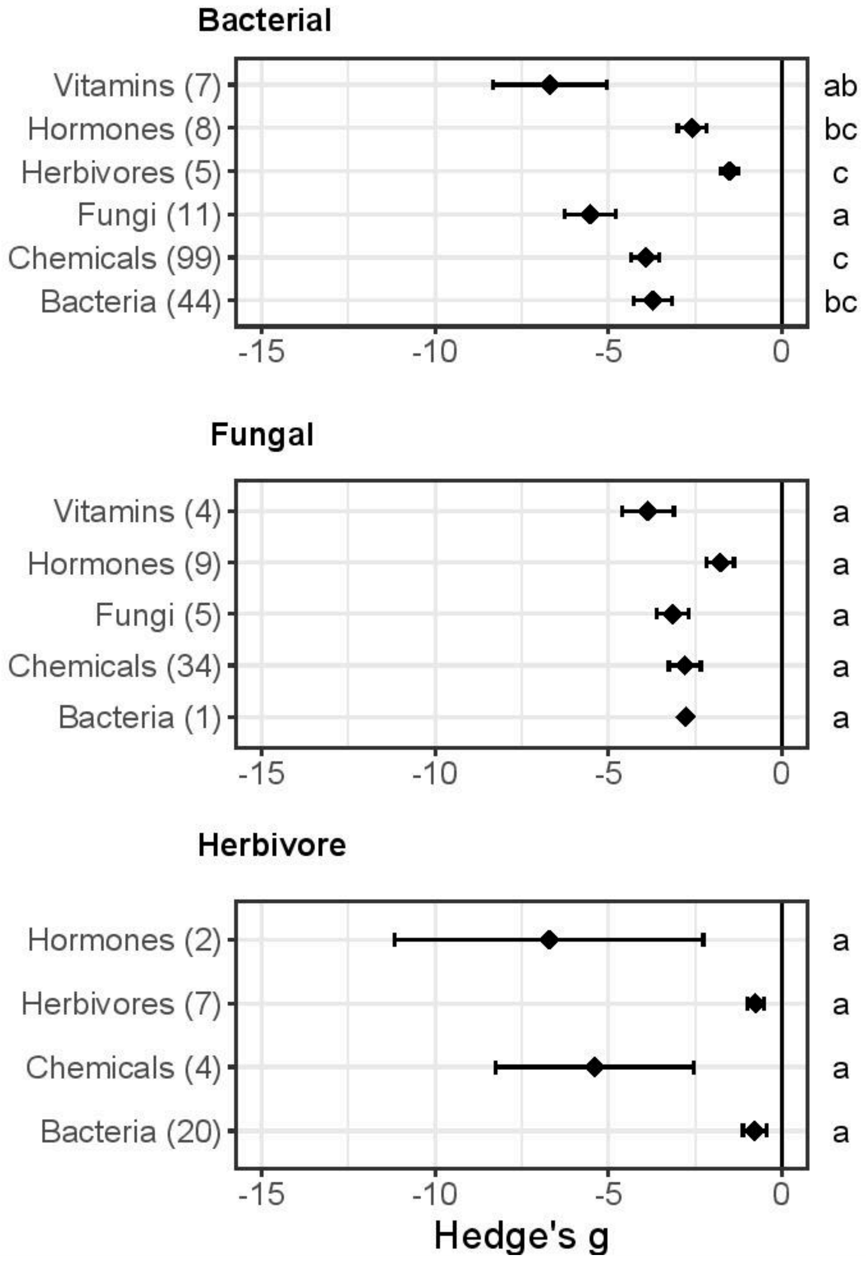

S2. Self-priming refers to priming of defences by an organism or group of organisms (here bacteria, fungi and herbivores) against the same kind of organism. a) Bacterial self-priming may provide less protection than fungal priming. b) Fungal self-priming provides no better protection against fungi than priming with bacteria, chemicals,

hormones or vitamins. c) Herbivore self-priming appears to be weak. Different letters along the right-hand axis indicate significant differences according to the Kruskal Wallis test (α 0.05) and subsequent Dunn's test (α 0.05). Negative values of Hedge's g ± SE indicate elevated resistance after priming. Sample sizes in brackets.

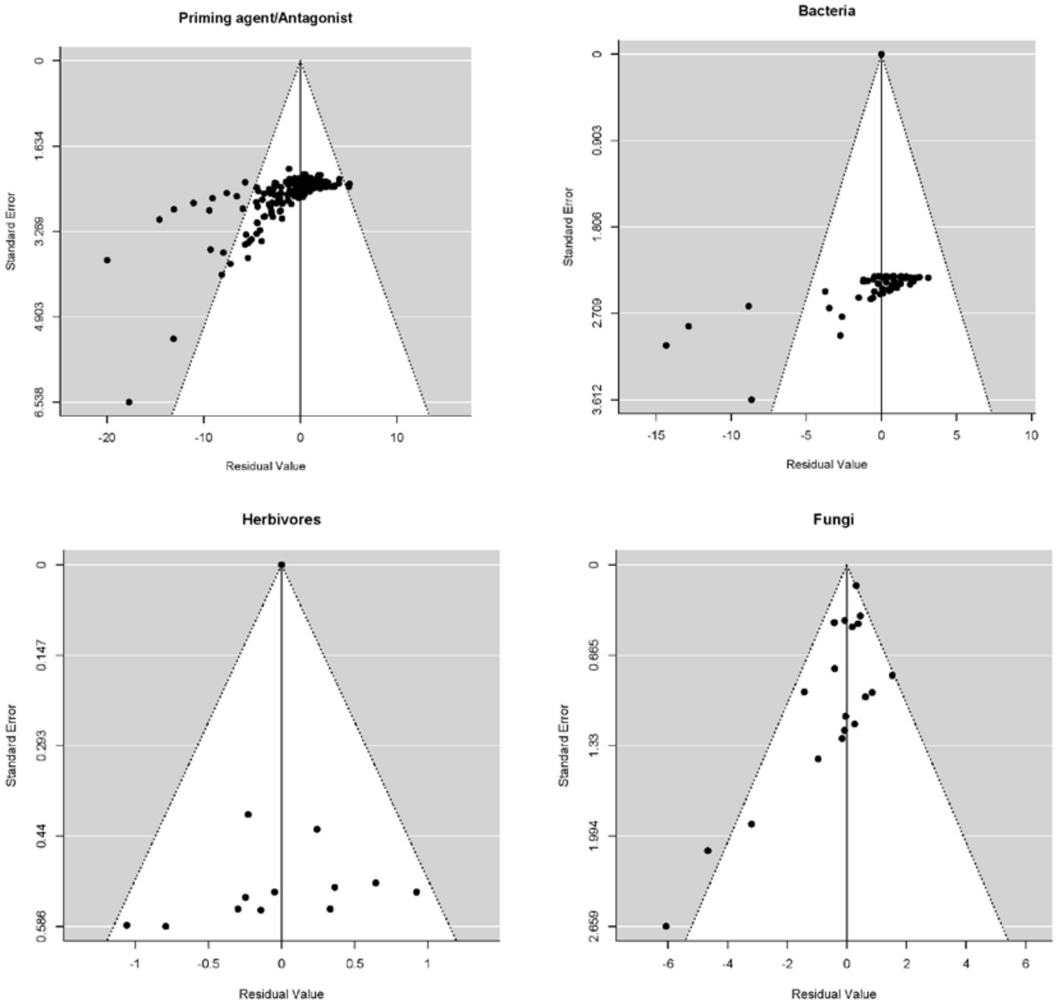

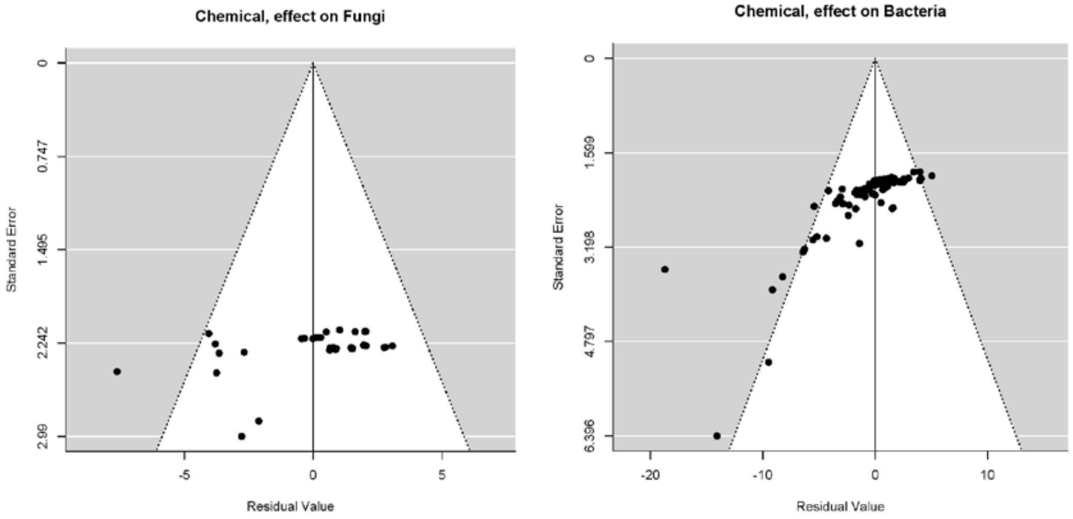

S3. Funnel plots assessing publication bias in the data. Upper legends indicate the data sets used to create each funnel plot (see Figs. 1-4), which were created using a Mixed Effect Model with Priming agents and stress agents as moderators. For more information about Funnel plots and Mixed Effect Models see Viechtbauer (2010)**.**

S4. Fail-safe numbers obtained using the Rosenthal approach (α 0.05), as implemented in the Metafor package in R. They indicate the number of non-significant findings required to reject the outcome of a meta-analysis. Fail-safe numbers greater than 5*N+10 indicate that the data are robust. N = number of experiments included in the meta-analysis (Rowen & Kaplan, 2016). The Priming agent column indicates the dataset used to calculate the Fail-safe number (see also Figs. 1-4).

**Priming agent**	**Fail-safe Number**	**N**
Bacteria	17081	65
Herbivores	689	13
Fungi	4022	20
Chemical, effect on Bacteria	33628	99
Chemical, effect on Fungi	11422	34
Priming Agent/Antagonist	356681	267

S5. Reference list for studies included in the meta-analysis.

Abe, H., Ohnishi, J., Narusaka, M., Seo, S., Narusaka, Y., Tsuda, S. and Kobayashi, M. Function of jasmonate in response and tolerance of *Arabidopsis* to thrip feeding. *Plant Cell Physiol.*, **49**, 68–80 (2008).

Ahn, I-P., Lee, S-W. and Suh, S-C. Rhizobacteria-Induced Priming in *Arabidopsis* Is Dependent on Ethylene, Jasmonic Acid, and *NPR1*. *Mol. Plant-Microbe Interact.*, **20**, 759–768. Available at: http://apsjournals.apsnet.org/doi/10.1094/MPMI-20-7-0759 (2007).

Ahn, I-P., Kim, S., Lee, Y-H. and Suh, S-C. Vitamin B1-Induced Priming Is Dependent on Hydrogen Peroxide and the *NPR1* Gene in Arabidopsis. *Plant Physiol.*, **143**, 838–848 (2007).

Attaran, E., Rostás, M. and Zeier, J. *Pseudomonas syringae* Elicits Emission of the Terpenoid (E,E)-4,8,12-Trimethyl-1,3,7,11-Tridecatetraene in Arabidopsis Leaves Via Jasmonate Signaling and Expression of the Terpene Synthase TPS4. *Mol. Plant-Microbe Interact.*, **21**, 1482–1497 (2008).

Aziz, M., Nadipalli, R.K., Xie, X., Sun, Y., Surowiec, K., Zhang, J-L. and Paré, P.W. Augmenting Sulfur Metabolism and Herbivore Defense in *Arabidopsis* by Bacterial Volatile Signaling. *Front. Plant Sci.*, **7**, 1–14. Available at: http://journal.frontiersin.org/Article/10.3389/fpls.2016.00458/abstract (2016).

Beckers, G.J.M., Jaskiewicz, M., Liu, Y., Underwood, W.R., He, S.Y., Zhang, S. and Conrath, U. Mitogen-Activated Protein Kinases 3 and 6 Are Required for Full Priming of Stress Responses in *Arabidopsis thaliana*. *Plant Cell Online*, **21**, 944–953. Available at: http://www.plantcell.org/cgi/doi/10.1105/tpc.108.062158 (2009).

Bernsdorff, F., Döring, A.-C., Gruner, K., Schuck, S., Bräutigam, A. and Zeier, J. Pipecolic acid orchestrates plant systemic acquired resistance and defense priming via salicylic acid-dependent and -independent pathways. *Plant Cell*, **28**, 102-129. Available at: http://www.plantcell.org/lookup/doi/10.1105/tpc.15.00496 (2016).

Canet, J.V., Dobón, A., Ibáñez, F., Perales, L. and Tornero, P. Resistance and biomass in Arabidopsis: a new model for Salicylic Acid perception. *Plant Biotechnol. J.*, **8**, 126–141 (2010).

Cartieaux, F., Contesto, C., Gallou, A., Desbrosses, G., Kopka, J., Taconnat, L., Renou, J-P., Touraine, B. Simultaneous Interaction of *Arabidopsis thaliana* with *Bradyrhizobium* Sp. Strain ORS278 and *Pseudomonas syringae* pv. *tomato* DC3000 Leads to Complex Transcriptome Changes. *Mol. Plant-Microbe Interact.*, **21**, 244-259 (2008).

Chaturvedi, R., Venables, B., Petros, R.A., Nalam, V., Li, M., Wang, X., Takemoto, L.J. and Shah, J. An abietane diterpenoid is a potent activator of systemic acquired resistance. *Plant J.*, **71**, 161–172 (2012).

Cho, S.M., Park, J.Y., Han, S.H., Anderson, A.J., Yang, K.Y., Gardener, B.M. and Kim, Y.C. Identification and transcriptional analysis of priming genes in *Arabidopsis thaliana* induced by root colonization with *Pseudomonas chlororaphis* O6. *Plant Pathol. J.*, **27**, 272–279 (2011).

Cordovez, V., Mommer, L., Moisan, K., Lucas-Barbosa, D., Pierik, R., Mumm, R., Carrion, V.J. and Raaijmakers, J.M. Plant Phenotypic and Transcriptional Changes Induced by Volatiles from the Fungal Root Pathogen Rhizoctonia solani. *Front. Plant Sci.*, **8**, 1–14. Available at: http://journal.frontiersin.org/article/10.3389/fpls.2017.01262/full (2017).

Davidsson, P., Broberg, M., Kariola, T., Sipari, N., Pirhonen, M. and Palva, E.T. Short oligogalacturonides induce pathogen resistance-associated gene expression in *Arabidopsis thaliana*. *BMC Plant Biol.*, **17**, 1–17. Available at: http://dx.doi.org/10.1186/s12870-016-0959-1 (2017).

De Vos, M., Van Zaanen, W., Koornneef, A., Korzelius, J.P., Dicke, M., Van Loon, L.C. and Pieterse, C.M.J. Herbivore-Induced Resistance against Microbial Pathogens in

Arabidopsis. *Plant Physiol.*, **142**, 352–363. Available at: http://www.plantphysiol.org/cgi/doi/10.1104/pp.106.083907 (2006).
De Vos, M. and Jander, G. *Myzus persicae* (green peach aphid) salivary components induce defence responses in *Arabidopsis thaliana*. *Plant, Cell Environ.*, **32**, 1548–1560 (2009).
Dellagi, A., Segond, D., Rigault, M., Fagard, M., Simon, C., Saindrenan, P. and Expert, D. Microbial Siderophores Exert a Subtle Role in Arabidopsis during Infection by Manipulating the Immune Response and the Iron Status. *Plant Physiol.*, **150**, 1687–1696. Available at: http://www.plantphysiol.org/cgi/doi/10.1104/pp.109.138636 (2009).
Deng, B., Deng, S., Sun, F., Zhang, S. and Dong, H. Down-regulation of free riboflavin content induces hydrogen peroxide and a pathogen defense in *Arabidopsis*. *Plant Mol. Biol.*, **77**, 185–201 (2011).
Dong, H-P., Yu, H., Bao, Z., Guo, X., Peng, J., Yao, Z., Chen, G., Qu, S. and Dong, H. The *ABI2*-dependent abscisic acid signalling controls HrpN-induced drought tolerance in *Arabidopsis*. *Planta*, **221**, 313–327 (2005).
Edgar, C.I., McGrath, K.C., Dombrecht, B., Manners, J.M., Maclean, D.C., Schenk, P.M. and Kazan, K. Salicylic acid mediates resistance to the vascular wilt pathogen *Fusarium oxysporum* in the model host *Arabidopsis thaliana*. *Australas. Plant Pathol.*, **35**, 581–591 (2006).
Elsharkawy, M.M., Shimizu, M., Takahashi, H. and Hyakumachi, M. Induction of systemic resistance against *Cucumber mosaic virus* by *Penicillium simplicissimum* GP17-2 in Arabidopsis and tobacco. *Plant Pathol.*, **61**, 964–976 (2012).
Elsharkawy, M.M., Shimizu, M., Takahashi, H., Ozaki, K. and Hyakumachi, M. Induction of systemic resistance against *Cucumber mosaic virus* in *Arabidopsis thaliana* by *Trichoderma asperellum* SKT-1. *Plant Pathol. J.*, **29**, 193–200 (2013).
Ferrari, S., Galletti, R., Denoux, C., De Lorenzo, G., Ausubel, F.M. and Dewdney, J. Resistance to *Botrytis cinerea* Induced in Arabidopsis by Elicitors Is Independent of Salicylic Acid, Ethylene, or Jasmonate Signaling But Requires PHYTOALEXIN DEFICIENT3. *Plant Physiol.*, **144**, 367–379. Available at: http://www.plantphysiol.org/cgi/doi/10.1104/pp.107.095596 (2007).
Gamir, J., Pastor, V., Kaever, A., Cerezo, M. and Flors, V. Targeting novel chemical and constitutive primed metabolites against *Plectosphaerella cucumerina*. *Plant J.*, **78**, 227–240 (2014).
García-Andrade, J., Ramírez, V., Flors, V. and Vera, P. Arabidopsis *ocp3* mutant reveals a mechanism linking ABA and JA to pathogen-induced callose deposition. *Plant J.*, **67**, 783–794 (2011).
Gupta, V., Willits, M.G. and Glazebrook, J. *Arabidopsis thaliana EDS4* Contributes to Salicylic Acid (SA)-Dependent Expression of Defense Responses: Evidence for Inhibition of Jasmonic Acid Signaling by SA. *Mol. Plant-Microbe Interact.*, **13**, 503–511. Available at: http://apsjournals.apsnet.org/doi/10.1094/MPMI.2000.13.5.503 (2000).
Harun-Or-Rashid, M., Khan, A., Hossain, M.T. and Chung, Y.R. Induction of Systemic Resistance against Aphids by Endophytic *Bacillus velezensis* YC7010 via Expressing *PHYTOALEXIN DEFICIENT4* in *Arabidopsis*. *Front. Plant Sci.*, **8**, 1–12. Available at: http://journal.frontiersin.org/article/10.3389/fpls.2017.00211/full (2017).
Hilfiker, O., Groux, R., Bruessow, F., Kiefer, K., Zeier, J. and Reymond, P. Insect eggs induce a systemic acquired resistance in Arabidopsis. *Plant J.*, **80**, 1085–1094 (2014).
Hossain, M.M., Sultana, F. and Hyakumachi, M. Role of ethylene signalling in growth and systemic resistance induction by the plant growth-promoting fungus *Penicillium viridicatum* in Arabidopsis. *J. Phytopathol.*, **165**, 432–441 (2017).
Hossain, M.M., Sultana, F., Kubota, M. and Hyakumachi, M. Differential inducible defense mechanisms against bacterial speck pathogen in *Arabidopsis thaliana* by plant-growth-promoting-fungus *Penicillium* sp. GP16-2 and its cell free filtrate. *Plant Soil*, **304**, 227–239 (2008).

Jiang, C.H., Huang, Z.Y., Xie, P., et al. Transcription factors WRKY70 and WRKY11 served as regulators in rhizobacterium *Bacillus cereus* AR156-induced systemic resistance to *Pseudomonas syringae* pv. tomato DC3000 in Arabidopsis. *J. Exp. Bot.*, **67**, 157–174 (2016).

Kawamura, Y., Takenaka, S., Hase, S., Kubota, M., Ichinose, Y., Kanayama, Y., Nakaho, K., Klessig, D.F. and Takahashi, H. Enhanced defense responses in Arabidopsis induced by the cell wall protein fractions from *Pythium oligandrum* require *SGT1*, *RAR1*, *NPR1* and *JAR1*. *Plant Cell Physiol.*, **50**, 924–934 (2009).

Kishimoto, K., Matsui, K., Ozawa, R. and Takabayashi, J. Components of C6-aldehyde-induced resistance in *Arabidopsis thaliana* against a necrotrophic fungal pathogen, *Botrytis cinerea*. *Plant Sci.*, **170**, 715–723 (2006).

Kishimoto, K., Matsui, K., Ozawa, R. and Takabayashi, J. Volatile C6-aldehydes and allo-ocimene activate defense genes and induce resistance against *Botrytis cinerea* in *Arabidopsis thaliana*. *Plant Cell Physiol.*, **46**, 1093–1102 (2005).

Kravchuk, Z., Vicedo, B., Flors, V., Camañes, G., González-Bosch, C. and García-Agustín, P. Priming for JA-dependent defenses using hexanoic acid is an effective mechanism to protect *Arabidopsis* against *B. cinerea*. *J. Plant Physiol.*, **168**, 359–366 (2011).

Lakshmanan, V., Castaneda, R., Rudrappa, T. and Bais, H.P. Root transcriptome analysis of *Arabidopsis thaliana* exposed to beneficial *Bacillus subtilis* FB17 rhizobacteria revealed genes for bacterial recruitment and plant defense independent of malate efflux. *Planta*, **238**, 657–668 (2013).

Li, B., Jiang, S., Yu, X., et al. Phosphorylation of Trihelix Transcriptional Repressor ASR3 by MAP KINASE4 Negatively Regulates Arabidopsis Immunity. *Plant Cell*, **27**, 839–856. Available at: http://www.plantcell.org/lookup/doi/10.1105/tpc.114.134809 (2015).

Li, F., Wang, J., Ma, C., Zhao, Y., Wang, Y., Hasi, A. and Qi, Z. Glutamate Receptor-Like Channel3.3 Is Involved in Mediating Glutathione-Triggered Cytosolic Calcium Transients, Transcriptional Changes, and Innate Immunity Responses in Arabidopsis. *Plant Physiol.*, **162**, 1497–1509. Available at: http://www.plantphysiol.org/cgi/doi/10.1104/pp.113.217208 (2013).

Mathys, J., De Cremer, K., Timmermans, P., Van Kerckhove, S., Lievens, B., Vanhaecke, M., Cammue, B.P.A. and De Coninck, B. Genome-Wide Characterization of ISR Induced in *Arabidopsis thaliana* by *Trichoderma hamatum* T382 Against *Botrytis cinerea* Infection. *Front. Plant Sci.*, **3**, 1–25. Available at: http://journal.frontiersin.org/article/10.3389/fpls.2012.00108/abstract (2012).

Mishina, T.E. and Zeier, J. Pathogen-associated molecular pattern recognition rather than development of tissue necrosis contributes to bacterial induction of systemic acquired resistance in Arabidopsis. *Plant J.*, **50**, 500–513 (2007).

Narusaka, Y., Narusaka, M., Abe, H., Hosaka, N., Kobayashi, M., Shiraishi, T. and Iwabuchi, M. High-throughput screening for plant defense activators using a *β-glucuronidase*-reporter gene assay in *Arabidopsis thaliana*. *Plant Biotechnol.*, **26**, 345–349 (2009).

Návarová H., Bernsdorff, F., Döring, A-C. and Zeier, J. Pipecolic Acid, an Endogenous Mediator of Defense Amplification and Priming, Is a Critical Regulator of Inducible Plant Immunity. *Plant Cell*, **24**, 5123–5141. Available at: http://www.plantcell.org/cgi/doi/10.1105/tpc.112.103564 (2012).

Niu, D-D., Liu, H-X., Jiang, C-H., Wang, Y-P., Wang, Q-Y., Jin, H-L. and Guo, J-H. The plant growth-promoting Rhizobacterium *Bacillus cereus* AR156 induces systemic resistance in *Arabidopsis thaliana* by simultaneously activating salicylate- and jasmonate/ethylene-dependent signaling pathways. *Mol. Plant. Microbe. Interact.*, **24**, 533–542 (2011).

Niu, D., Xia, J., Jiang, C., et al. *Bacillus cereus* AR156 primes induced systemic resistance by suppressing miR825/825* and activating defense-related genes in *Arabidopsis*. *J. Integr. Plant Biol.*, **58**, 426–439 (2016).

Noh, S.W., Seo, R., Park, J-K., Manir, M.M., Park, K., Sang, M.K., Moon, S-S. and Jung, H.W. Cyclic dipeptides from *Bacillus vallismortis* BS07 require key components of plant immunity to induce disease resistance in *Arabidopsis* against *Pseudomonas* infection. *Plant Pathol. J.*, **33**, 402–409 (2017).

Pangesti, N., Pineda, A., Dicke, M. and Van Loon, J.J.A. Variation in plant-mediated interactions between rhizobacteria and caterpillars: potential role of soil composition. *Plant Biol.*, **17**, 474–483 (2015).

Pastor, V., Balmer, A., Gamir, J., Flors, V. and Mauch-Mani, B. Preparing to fight back: generation and storage of priming compounds. *Front. Plant Sci.*, **5**, 1–12. Available at: http://journal.frontiersin.org/article/10.3389/fpls.2014.00295/abstract (2014).

Pastor, V., Luna, E., Ton, J., Cerezo, M., García-Agustín, P. and Flors, V. Fine tuning of reactive oxygen species homeostasis regulates primed immune responses in *Arabidopsis*. *Mol. Plant. Microbe. Interact.*, **26**, 1334–1344. Available at: http://www.ncbi.nlm.nih.gov/pubmed/24088017 (2013).

Pieterse, C.M.J., Van Pelt, J.A., Ton, J., Parchmann, S., Mueller, M.J., Buchala, A.J., Métraux, J-P. and Van Loon, L.C. Rhizobacteria-mediated induced systemic resistance (ISR) in *Arabidopsis* requires sensitivity to jasmonate and ethylene but is not accompanied by an increase in their production. *Physiol. Mol. Plant Pathol.*, **57**, 123–134 (2000).

Pineda, A., Zheng, S-J., Van Loon, J.J.A. and Dicke, M. Rhizobacteria modify plant-aphid interactions: a case of induced systemic susceptibility. *Plant Biol.*, **14**, 83–90 (2012).

Po-Wen, C., Singh, P. and Zimmerli, L. Priming of the Arabidopsis pattern-triggered immunity response upon infection by necrotrophic *Pectobacterium carotovorum* bacteria. *Mol. Plant Pathol.*, **14**, 58–70 (2013).

Raacke, I.C., Von Rad, U., Mueller, M.J. and Berger, S. Yeast Increases Resistance in Arabidopsis Against *Pseudomonas syringae* and *Botrytis cinerea* by Salicylic Acid – Dependent as Well as – Independent Mechanisms. *Mol. Plant. Microbe. Interact*, **19**, 1138–1146 (2006).

Scala, A., Mirabella, R., Mugo, C., Matsui, K., Haring, M.A. and Schuurink, R.C. *E*-2-hexenal promotes susceptibility to Pseudomonas syringae by activating jasmonic acid pathways in *Arabidopsis*. *Front. Plant Sci.*, **4**, 1–11. Available at: http://journal.frontiersin.org/article/10.3389/fpls.2013.00074/abstract (2013).

Schenk, S.T., Hernandez-Reyes, C., Samans, B., et al. *N*-Acyl-Homoserine Lactone Primes Plants for Cell Wall Reinforcement and Induces Resistance to Bacterial Pathogens via the Salicylic Acid/Oxylipin Pathway. *Plant Cell*, **26**, 2708–2723. Available at: http://www.plantcell.org/cgi/doi/10.1105/tpc.114.126763 (2014).

Schreiber, K., Ckurshumova, W., Peek, J. and Desveaux, D. A high-throughput chemical screen for resistance to *Pseudomonas syringae* in Arabidopsis. *Plant J.*, **54**, 522–531 (2008).

Singh, V., Roy, S., Giri, M.K., Chaturvedi, R., Chowdhury, Z., Shah, J. and Nandi, A.K. *Arabidopsis thaliana FLOWERING LOCUS D* Is Required for Systemic Acquired Resistance. *Mol. Plant-Microbe Interact.*, **26**, 1079–1088. Available at: http://apsjournals.apsnet.org/doi/10.1094/MPMI-04-13-0096-R (2013).

Song, G.C., Choi, H.K. and Ryu, C-M. Gaseous 3-pentanol primes plant immunity against a bacterial speck pathogen, *Pseudomonas syringae* pv. tomato via salicylic acid and jasmonic acid-dependent signaling pathways in *Arabidopsis*. *Front. Plant Sci.*, **6**, 1–7. Available at: http://journal.frontiersin.org/Article/10.3389/fpls.2015.00821/abstract (2015).

Stahl, E., Bellwon, P., Huber, S., Schlaeppi, K., Bernsdorff, F., Vallat-Michel, A., Mauch, F. and Zeier, J. Regulatory and Functional Aspects of Indolic Metabolism in Plant Systemic Acquired Resistance. *Mol. Plant*, **9**, 662–681 (2016).

Suárez, L., Savatin, D. V., Salvi, G., De Lorenzo, G., Cervone, F. and Ferrari, S. The non-traditional growth regulator pectimorf is an elicitor of defense responses and protects Arabidopsis against *Botrytis cinerea*. *J. Plant Pathol.*, **95**, 177–180 (2013).

Subramanian, S., Sangha, J.S., Gray, B.A., Singh, R.P., Hiltz, D., Critchley, A.T. and Prithiviraj, B. Extracts of the marine brown macroalga, *Ascophyllum nodosum*, induce jasmonic acid dependent systemic resistance in *Arabidopsis thaliana* against *Pseudomonas syringae* pv. *tomato* DC3000 and *Sclerotinia sclerotiorum*. *Eur. J. Plant Pathol.*, **131**, 237–248 (2011).

Sultana, F., Hossain, M.M., Kubota, M. and Hyakumachi, M. Induction of systemic resistance in *Arabidopsis thaliana* in response to a culture filtrate from a plant growth-promoting fungus, *Phoma* sp. GS8-3. *Plant Biol.*, **11**, 97–104 (2009).

Thomma, B.P.H.J., Eggermont, K., Broekaert, W.F. and Cammue, B.P.A. Disease development of several fungi on Arabidopsis can be reduced by treatment with methyl jasmonate. *Plant Physiol. Biochem.*, **38**, 421–427 (2000).

Tjamos, S.E., Flemetakis, E., Paplomatas, E.J. and Katinakis, P. Induction of Resistance to *Verticillium dahliae* in *Arabidopsis thaliana* by the Biocontrol Agent K-165 and Pathogenesis-Related Proteins Gene Expression. *Mol. Plant-Microbe Interact.*, **18**, 555–561. Available at: http://apsjournals.apsnet.org/doi/10.1094/MPMI-18-0555 (2005).

Ton, J., Jakab, G., Toquin, V., Flors, V., Iavicoli, A., Maeder, M.N., Metrauxt, J-P. and Mauch-Mani, B. Dissecting the β-aminobutyric acid–induced priming phenomenon in Arabidopsis. *Plant J.*, **17**, 987–999 (2005).

Ton, J. and Mauch-Mani, B. β-amino-butyric acid-induced resistance against necrotrophic pathogens is based on ABA-dependent priming for callose. *Plant J.*, **38**, 119–130 (2004).

Van der Ent, S., Van Hulten, M., Pozo, M.K., Czechowski, T., Udvardi, M., Pieterse, C.M.J. and Ton, J. Priming of plant innate immunity by rhizobacteria and beta-aminobutyric acid: differences and similarities in regulation. *New Phytol.*, **183**, 419–431. Available at: http://www.ncbi.nlm.nih.gov/pubmed/19413686 (2009).

Van Oosten, V.R., Bodenhausen, N., Reymond, P., Van Pelt, J.A, Van Loon, L.C., Dicke, M. and Pieterse, C.M.J. Differential Effectiveness of Microbially Induced Resistance Against Herbivorous Insects in *Arabidopsis*. *Mol. Plant-Microbe Interact.*, **21**, 919–930. Available at: http://apsjournals.apsnet.org/doi/10.1094/MPMI-21-7-0919 (2008).

Vos, I.A., Verhage, A., Schuurink, R.C., Watt, L.G., Pieterse, C.M.J. and Wees, S.C.M. Van Onset of herbivore-induced resistance in systemic tissue primed for jasmonate-dependent defenses is activated by abscisic acid. *Front. Plant Sci.*, **4**, 1–10. Available at: http://journal.frontiersin.org/article/10.3389/fpls.2013.00539/abstract (2013).

Wang, X., Basnayake, B.M.V.S., Zhang, H., Li, G., Li, W., Virk, N., Mengiste, T. and Song, F. The *Arabidopsis* ATAF1, a NAC Transcription Factor, Is a Negative Regulator of Defense Responses Against Necrotrophic Fungal and Bacterial Pathogens. *Mol. Plant-Microbe Interact.*, **22**, 1227–1238. Available at: http://apsjournals.apsnet.org/doi/10.1094/MPMI-22-10-1227 (2009).

Wu, C.C., Singh, P., Chen, M.C. and Zimmerli, L. L-Glutamine inhibits beta-aminobutyric acid-induced stress resistance and priming in *Arabidopsis*. *J. Exp. Bot.*, **61**, 995–1002 (2010).

Yasuda, M., Ishikawa, A., Jikumaru, Y., et al. Antagonistic Interaction between Systemic Acquired Resistance and the Abscisic Acid-Mediated Abiotic Stress Response in *Arabidopsis*. *Plant Cell Online*, **20**, 1678–1692. Available at: http://www.plantcell.org/cgi/doi/10.1105/tpc.107.054296 (2008).

Yeh, Y-H., Panzeri, D., Kadota, Y., et al. The Arabidopsis Malectin-Like/LRR-RLK IOS1 is Critical for BAK1-Dependent and BAK1-Independent Pattern-Triggered Immunity. *Plant Cell*, **28**, 1701-1721. Available at: http://www.plantcell.org/lookup/doi/10.1105/tpc.16.00313 (2016).

Zahid, A., Jaber, R., Laggoun, F., et al. Holaphyllamine, a steroid, is able to induce defense responses in *Arabidopsis thaliana* and increases resistance against bacterial infection. *Planta*, **246**, 1109–1124 (2017).

Zhang, C., Shi, H., Chen, L., et al. Harpin-induced expression and transgenic overexpression of the phloem protein gene *AtPP2-A1* in Arabidopsis repress phloem feeding of the green peach aphid *Myzus persicae*. *BMC Plant Biol.*, **11**, 1-19. Available at: http://www.biomedcentral.com/1471-2229/11/11 (2011).

Zhang, H. *Arabidopsis* AtERF15 positively regulates immunity against *Pseudomonas syringae* pv. *tomato* DC3000 and *Botrytis cinerea*. *Front. Plant Sci.*, **6**, 1–13. Available at: http://journal.frontiersin.org/article/10.3389/fpls.2015.00686/full (2015).

Zhang, S., Yang, X., Sun, M., Sun, F., Deng, S. and Dong, H. Riboflavin-induced priming for pathogen defense in *Arabidopsis thaliana*. *J. Integr. Plant Biol.*, **51**, 167–174 (2009).

Zhou, J., Sun, A. and Xing, D. Modulation of cellular redox status by thiamine-activated NADPH oxidase confers *Arabidopsis* resistance to *Sclerotinia sclerotiorum*. *J. Exp. Bot.*, **64**, 3261–3272 (2013).

Zhou, N., Tootle, T.L., Tsui, F., Klessig, D.F. and Glazebrook, J. PAD4 Functions Upstream from Salicylic Acid to Control Defense Responses in Arabidopsis. *Plant Cell*, **10**, 1021-1030. Available at: http://www.jstor.org/stable/10.2307/3870687?origin=crossref (1998).

S6. Added references to Table 1.

Babu, A., Jogaiah, S., Ito, S.I., Nagaraj, A.K. & Tran, L.S.P. Improvement of growth, fruit weight and early blight disease protection of tomato plants by rhizosphere bacteria is correlated with their beneficial traits and induced biosynthesis of antioxidant peroxidase and polyphenol oxidase. Plant Sci. **231**, 62–73 (2015).

Berglund, T., Lindstrom, A., Aghelpasand, H., Stattin, E. & Ohlsson, A.B. Protection of spruce seedlings against pine weevil attacks by treatment of seeds or seedlings with nicotinamide, nicotinic acid and jasmonic acid. Forestry **89**, 127-135 (2016).

Castillo Lopez, D., Zhu-Salzman, K., Ek-Ramos, M.J. & Sword, G.A. The entomopathogenic fungal endophytes Purpureocillium lilacinum (formerly Paecilomyces lilacinus) and Beauveria bassiana negatively affect cotton aphid reproduction under both greenhouse and field conditions. PLoS One **9**, e103891 (2014).

Hamada, A.M., Fatehi, J. & Jonsson, L.M.V. Seed treatments with thiamine reduce the performance of generalist and specialist aphids on crop plants. Bulletin of Entomological Research, **108**, 84-92 (2018).

Hamada, A.M. & Jonsson, L.M.V. Thiamine treatments alleviate aphid infestations in barley and pea. Phytochemistry **94**, 135-141, doi:10.1016/j.phytochem.2013.05.012 (2013).

Jogaiah, S., Abdelrahman, M., Tran, L.S.P. & Shin-Ichi, I. Characterization of rhizosphere fungi that mediate resistance in tomato against bacterial wilt disease. J. Exp. Bot. **64**, 3829–3842 (2013).

Nagaraju, A., Sudisha, J. & Mahadeva Murthy, S. Seed priming with Trichoderma harzianum isolates enhances plant growth and induces resistance against Plasmopara halstedii, an incitant of sunflower downy mildew disease. Austalasian Plant Pathol **41**, 609–620 (2012).

Pushpalatha, H.G., Mythrashree, S.R., Shetty, R., Geetha, N.P., Sharathchandra, R.G., Amruthesh, K.N. & Shetty, H.S. Ability of vitamins to induce downy mildew disease resistance and growth promotion in pearl millet. Crop Protection **26**, 1674-1681, doi:10.1016/j.cropro.2007.02.012 (2007).

Pushpalatha, H.G., Sudisha, J., Geetha, N.P., Amruthesh, K.N. & Shetty, H.S. Thiamine seed treatment enhances LOX expression, promotes growth and induces downy mildew disease resistance in pearl millet. Biologia Plantarum **55**, 522-527, doi:10.1007/s10535-011-0118-3 (2011).

Song, G.C., Choi, H.K., Kim, Y.S., Choi, J.S. & Ryu, C.M. Seed defense biopriming with bacterial cyclodipeptides triggers immunity in cucumber and pepper. Scientific Reports **7**, doi:ARTN 14209 (2017).

Worrall, D., Holroyd, G.H., Moore, J.P., Glowacz, M., Croft, P., Taylor, J.E., Paul, N.D. & Roberts, M.R. Treating seeds with activators of plant defence generates long-lasting priming of resistance to pests and pathogens. New Phytologist, **193**, 770-778, doi:10.1111/j.1469-8137.2011.03987.x (2012).
